# Dermatomyositis-Related Encephalopathy: Clinical, Neuroimaging and Immunological Characterization

**DOI:** 10.3390/diagnostics15060700

**Published:** 2025-03-12

**Authors:** Daniel Alberto Carrillo-Vázquez, Carlos Antonio Davizon-López, Alejandro Gutiérrez-Castillo, Jiram Torres-Ruiz, Alfredo Pérez-Fragoso, Beatriz Alcalá-Carmona, Alejandro Barrera-Godínez, Guillermo Juárez-Vega, Lidia Antonia Gutiérrez-Gutiérrez, Rodrigo Hernández-Ramírez, Diana Gómez-Martín

**Affiliations:** 1 Department of Internal Medicine, Instituto Nacional de Ciencias Médicas y Nutrición Salvador Zubirán, Mexico City 14080, Mexico; danielbeatle94@gmail.com (D.A.C.-V.); carlos.davizon25@gmail.com (C.A.D.-L.); alejandro.gutierrezc@incmnsz.mx (A.G.-C.); 2 Department of Immunology and Rheumatology, Instituto Nacional de Ciencias Médicas y Nutrición Salvador Zubirán, Mexico City 14080, Mexico; josetorresruiz85@gmail.com (J.T.-R.); peralfra07@gmail.com (A.P.-F.); beatriz.alcalac@gmail.com (B.A.-C.); 3 Department of Dermatology, Instituto Nacional de Ciencias Médicas y Nutrición Salvador Zubirán, Mexico City 14080, Mexico; alejandro.barrera.g@gmail.com; 4 Red de Apoyo a la Investigación, Coordinación de Investigación Científica, Universidad Nacional Autónoma de México, Mexico City 14080, Mexico; guillermovega@cic.unam.mx; 5 Department of Neurology and Neuropsychology, Instituto Nacional de Ciencias Médicas y Nutrición Salvador Zubirán, Mexico City 14080, Mexico; lidia.gutierrezg@incmnsz.mx; 6 Department of Nuclear Medicine, Instituto Nacional de Ciencias Médicas y Nutrición Salvador Zubirán, Mexico City 14080, Mexico; rodrigo.hernandezr@incmnsz.mx

**Keywords:** dermatomyositis, encephalopathy, immunophenotype

## Abstract

**Background/Objectives**: Dermatomyositis (DM) is an autoimmune disease with rarely reported central nervous system involvement, such as encephalopathy. However, no objective characterization of dermatomyositis patients with neurocognitive decline has been previously addressed. **Methods**: Herein, we describe the immunophenotype, clinical, and neuroimaging features of three DM patients with encephalopathy. **Results**: The neurocognitive profile of the three patients was characterized by abnormalities in attention, working memory, and language. PET/CT demonstrated temporal and occipital cortical hypometabolism with hypermetabolism in the mesial temporal region, cerebellar, and basal nuclei. The peripheral immunophenotype of DM patients with encephalopathy demonstrated enhanced expression of PD-1+ in CD4+ and CD8+ T cells in comparison with DM patients without encephalopathy. In comparison to healthy controls, DM patients with encephalopathy had increased naïve CD4+, CD57+, and CD4+ T cells, effector memory (TEM), and CD73+ and CD8+ T cells. Additionally, the normalization of cerebral metabolism and clinical behavior after immunosuppressive treatment was evidenced. **Conclusions**: The PET/CT profile and peripheral immunophenotype (PD-1+, TEM, CD57+, and CD73+) could help to recognize DM patients who are prone to developing encephalopathy symptoms in order to avoid sequelae.

## 1. Introduction

Dermatomyositis is a subgroup of systemic autoimmune diseases named idiopathic inflammatory myopathies (IIM) that involves predominantly proximal muscle weakness, dysphagia, cutaneous lesions, and extra muscular organ damage such as lung, skin, and joints [1,2]. Several clinicopathologic subtypes have been identified: dermatomyositis (DM), polymyositis (PM), immune-mediated necrotizing myopathy, sporadic inclusion body myositis, and myositis overlap with other autoimmune conditions. DM is highly characterized by vasculopathy features, such as diffuse interstitial lung disease (ILD), calcinosis, ulcers, and hyperkeratotic plaques (mechanic’s hands or hiker’s feet) [1,3]. Autoimmune encephalopathy is a rare manifestation of systemic autoimmune diseases such as dermatomyositis [4]. Major advances in neuroimaging and immunotherapy have improved the awareness of these entities; however, no clinical, neuroimaging, or immunological characterization of patients with dermatomyositis with neurocognitive decline has been addressed to this date. Recently, extrafollicular response, T effector memory (CD45RO^+^CCR7^+^), immunosenescence, and suppression-related markers such as CD57^+^, CD73^+^, and PD-1^+^ have been correlated with poor prognosis, including high morbidity and mortality in autoimmune diseases [5,6,7,8].

Here, we describe the clinical, neuroimaging, and immunological features of three patients with a previously unrecognized association of DM complicated by cognitive impairment.

## 2. Materials and Methods

Study design: We performed an observational cross-sectional analytical study comparing the clinical, imaging, and pathological characteristics of DM patients with encephalopathy to those of DM patients without encephalopathy.

Study setting: We prospectively recruited nine patients hospitalized at the Instituto Nacional de Ciencias Médicas y Nutrición Salvador Zubirán (a tertiary care center in Mexico) for DM confirmed by muscle biopsy findings and 2017 ACR/EULAR IIM classification criteria [9].

Study period: The recruitment period of this study started on January 18th of 2022 and finished on August 9th of 2023.

Study participants: As disease controls, three patients had active disease by manual muscle test 8 (MMT8) <136 and/or cutaneous dermatomyositis disease area and severity index (CDASI) >6. Three were considered in complete clinical response/remission. The three patients with DM and encephalopathy had cognitive impairment, with a Montreal Cognitive Assessment (MoCA) screening test score <26; one had possible autoimmune encephalitis according to the Graus’ criteria [10]. As a negative control group, we included six age- and sex-matched healthy subjects. The active DM group was immunotherapy-matched with the DM and encephalopathy patients.

All three patients with DM-related encephalopathy were included to perform cognitive evaluation, Neuro PET/CT protocol, and peripheral immunophenotype at the time of inclusion and 3 months post-treatment follow-up.

Data collection: Two certified rheumatologists (J.T.R. and D.G.M.) evaluated the disease activity using the MMT8 and CDASI. LGG, a certified neuropsychologist, performed the neurocognitive evaluations, and RHR, a radiologist, certified nuclear medicine doctor, and specialist in Neuro PET/CT image interpretation, performed the semiquantitative and qualitative analysis of images. Exclusion criteria included acute or chronic infection, pregnancy, puerperium, and active neoplasia. All patients and healthy controls signed a written informed consent before inclusion; the protocol was approved by our Institutional Ethics and Research Committees (Ref. 2984) in compliance with the Helsinki Declaration. We collected a venous blood sample from patients with DM and healthy controls in the morning and after a 12-h fasting period. The samples were obtained before plasmapheresis, IVIg, and Rituximab administration. Myositis-specific antibodies (MSA) were assayed using Immunoblot (Euroimmune AG, Lübeck, Germany). The extended panels of surface autoimmune encephalitis antibodies (anti-NMDAR, AMPAR, GABABR, GABAAR, LGI1, CASPR2, mGluR1, mGluR2, mGluR5, DPPX, IgLON5, Neurexin) were performed in CSF as previously described [11]; briefly, tissue-based immunohistochemistry (IHC) of cellular surface in rat brain with the avidin-biotin-peroxidase complex method was performed and certified by the Center of Neuroimmunology and Paraneoplastic Disorders (Synlab, Barcelona, Spain).

### Operational Definitions

Active dermatomyositis: Patients that met the ACR/EULAR 2017 criteria with a muscle biopsy compatible and had either a manual muscle test 8 (MMT8) <136 and/or cutaneous dermatomyositis disease area and severity index (CDASI) >6.

Complete clinical response dermatomyositis: Patients that met the ACR/EULAR 2017 criteria with a muscle biopsy compatible and had either manual muscle test 8 (MMT8) >136 and cutaneous dermatomyositis disease area and severity index (CDASI) <6.

Dermatomyositis with encephalopathy: Active or complete clinical response patients with cognitive impairment, compatible with a Montreal Cognitive Assessment (MoCA) screening test score <26; one had possible autoimmune encephalitis according to the Graus’ criteria.

Cognitive Evaluation: In the three patients with cognitive impairment (MoCA score <26) [12], a broader neuropsychological assessment was performed using the NEUROPSI, an abbreviated neuropsychologic battery, which includes high-validity neuropsychologic tests standardized for Spanish-speaking Latin Americans. NEUROPSI briefly assesses a wide spectrum of cognitive functions, including orientation, attention, memory, language, visuoperceptual abilities, and executive functions. Application time is about 25 min, and the test is concise and reliable, providing initial or predictive diagnoses of cognitive alterations in populations with different levels of schooling, including illiterate people. The rating system provides qualitative and quantitative data. With the independent data of each cognitive ability, an individual profile is obtained, which indicates the abilities and inabilities of the subject in each one of the evaluated areas [13].

PET/CT protocol: ^18^F-FDG PET included a brain and whole-body scan, which was performed with a PET/CT Discovery 710 (GE Healthcare, Chicago, IL, USA). A radiologist and a nuclear medicine specialist performed semiquantitative and qualitative analyses of the images. For the visual analysis, intensity normalization was performed on the primary sensorimotor cortex. For the semiquantitative analysis, we used CortexID software by GE Healthcare, Chicago, IL, USA, which was also normalized to the global cortex. Automatic analyses were made as a comparison with the corresponding tracer uptake between our patients and normal subjects, getting a Z-score. According to previous studies, we selected brain regions to analyze (frontal lobe, temporal lateral region, temporal mesial region, occipital lobe, and cerebellum) [14,15]. Negative Z scores indicated hypometabolism and positive Z scores indicated hypermetabolism (cutoff of -2 and +2). No antipsychotic, sedative-hypnotics or neuroleptic drugs were administered before the scan.

Immunophenotype of peripheral blood mononuclear cells (PBMCs): PBMCs were isolated by a density gradient using Ficoll-Paque (GE Healthcare Life Sciences, Chicago, IL, USA). The cells were washed twice with phosphate-buffered saline (PBS) and incubated with the viability marker Zombie Aqua (Biolegend, San Diego, CA, USA). After two washes with staining buffer (5% fetal bovine serum in PBS), the cells were stained for 30 min at room temperature (RT) with the following fluorochrome-coupled antibodies: CD3-APC/Fire-750, CD4-Alexa Fluor 488, CD8-PE/Dazzle-594, CD45RA-PE/Cy7, CD45RO-PerCP, CD62L-PE, CCR7-Alexa Fluor 700, PD-1-APC, CD57-BV785, CD73-BV711 (all from Biolegend). A 4-laser LSR Fortessa flow cytometer (BD Biosciences,San Jose, CA, USA) was used to acquire 1 million events per sample.

The following lymphocyte subsets were characterized: CD4^+^ T cells (CD3^+^CD4^+^), CD8^+^ T cells (CD3^+^CD8^+^), naïve T cells (CD3^+^CD4^+^ or CD8^+^CD45RA^+^CD45RO^−^), memory T cells (CD3^+^CD4^+^ or CD8^+^CD45RA^−^CD45RO^+^), central memory T cells (CD3^+^CD4^+^ or CD8^+^CD45RA^−^CD45RO^+^CD62L^+^CCR7^+^), effector memory T cells (CD3^+^CD4^+^ or CD8^+^CD45RA^−^CD45RO^+^CD62L^−^CCR7^−^), PD-1^+^ T cells (CD3^+^CD4^+^ or CD8^+^PD-1^+/hi^), CD57^+^ T cells (CD3^+^CD4^+^ or CD8^+^CD57^+^), and CD73^+^ T cells (CD3^+^CD4^+^ or CD8^+^CD73^+^). The absolute number of T cell subsets was calculated according to the total lymphocyte count in a blood sample drawn on the same day. Expression of PD1, CD57, and CD73 was also assessed by mean fluorescence intensity (MFI). The samples were analyzed using FlowJo v10.7 software (BD Biosciences).

Statistical Analysis: Due to the non-parametric distribution of quantitative variables, they were expressed as median with interquartile range (IQR) or absolute numbers with percentage (%). The non-parametric data were analyzed with the Kruskall–Wallis test and the adjustment was made using the Dunn multiple comparison test, as well as the Mann–Whitney U test (sum of Wilcoxon ranges) to compare medians between groups. GraphPad Prism software, 10th version (La Jolla, CA, USA) was used to perform statistical analysis and graphics visualization. *p* values < 0.05 were considered significant.

## 3. Results

### 3.1. Main Clinical and Laboratory Features of Patients with Dermatomyositis with and Without Encephalopathy

**Figure 1:**
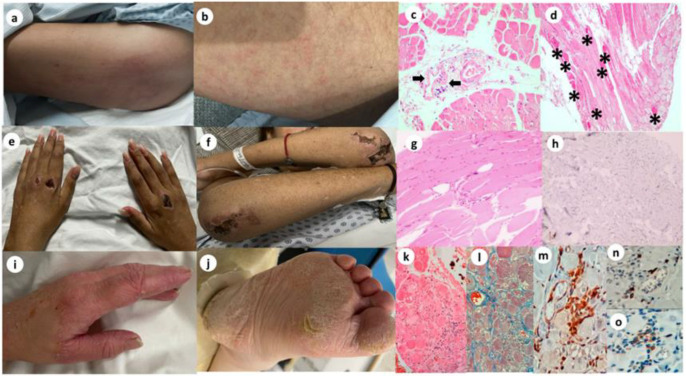
Clinical features of dermatomyositis patients with autoimmune encephalopathy corroborated with typical findings from muscle biopsy. Anti-TIF1-γ dermatomyositis and autoimmune encephalopathy: (**a**,**b**) Diffuse erythematous-purpuric rash and Gottron’s sign in thighs and forearms. Panoramic view of cross-sections showing areas with atrophy of the perifascicular musculoskeletal fibers, with decreased diameter in relation to the adjacent muscle fibers (H&E stain, 10×): (**c**) Focal inflammatory infiltrate, with perivascular and permissial lymphocytes (black arrows). (**d**) (H&E stain, 10×): Necrotic and regenerating muscle fibers (eosinophilic fibers*). anti-MDA5 dermatomyositis and anti-neuronal autoimmune encephalopathy: (**e**) necrotized ulcerative sores with erythematosus retiform well-defined borders in metacarpophalangeal joints. (**f**) ulcerative lesions in elbows. (**g**) (H&E stain, 20×): muscle fiber sizes altered with mild perifascicular atrophy and focus of a scarce inflammatory component (lymphocytes) evident in the perimysium and endomysium. (**h**) (IHQ CD4, 20×): focal immunohistochemistry positivity for the lymphocytes. anti-Mi2 dermatomyositis with thrombotic microangiopathy and anti-neuronal autoimmune encephalitis: (**i**) Mechanic hands. (**j**) Hiker feet. (**k**) (H&E stain 40×): Vacuolar degeneration of muscle fibers with peri- and endomysial inflammatory infiltrate. (**l**) (Mason stain 40×): Inflammatory infiltrate in perimysium and endomysium with collagen deposits. (**m**) (IHQ CD68 40×): Macrophage positivity. (**n**) (IHQ CD20 40×): B lymphocyte positivity. (**o**) (IHQ CD4 40×): T lymphocyte positivity.

The main demographic, clinical, and laboratory features are shown in Table 1. All the DM patients with encephalopathy had proximal and axial weakness, dysphagia, and dysphonia. The median manual muscle test 8 (MMT8) was 96 (80–120) (Table 1). The myositis-specific antibodies (MSA) were anti-transcription intermediate factor 1-gamma (TIF1-γ), anti-melanoma differentiation-associated gene 5 (MDA5), and Mi-2. The anti-TIF1-γ positive patient had Gottron’s papules on physical examination (Figure 1a,b). The muscle biopsy revealed perifascicular atrophy with acute inflammation (Figure 1c,d). On physical examination, the anti-MDA5 positive patient had a cutaneous rash, including ulcerated Gottron’s papules and poikiloderma (Figure 1e,f). The histopathological exam showed perifascicular inflammation, atrophy, and fatty infiltration with endomysial fibrosis (Figure 1g,h). The anti-Mi2 positive patient had mechanic’s hands and hiker’s feet (Figure 1i,j). Muscle biopsy showed perifascicular atrophy, infiltrating CD4+, macrophages, and B lymphocytes (Figure 1k–o).

### 3.2. Main Clinical and Laboratory Features of Patients with Dermatomyositis with Encephalopathy

**Figure 2:**
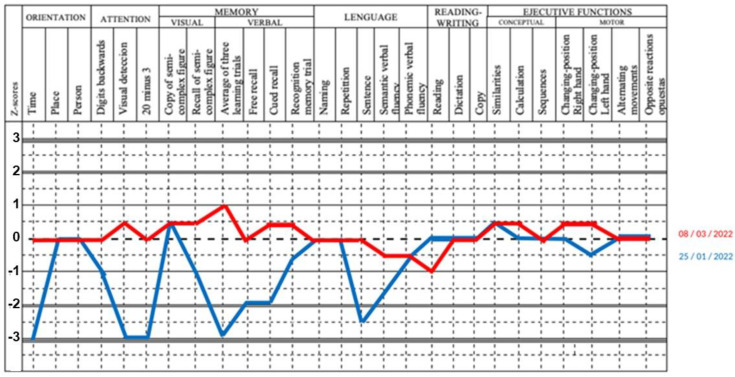
Basal and post-treatment neuropsychology assessment of patient #1. NEUROPSI modified the profile from Ostrosky’s original of the first patient (blue line), integrating mild slowing of thought, impulsiveness, and over-elaboration of verbal responses compatible with multiple domain amnesic neurocognitive impairment and cortico-subcortical compromise. After a 3-month follow-up, he improved his attention, visual and verbal memory, language emission, fluency with cognitive recovery, greater emotional control, and behavioral awareness (red line).

**Figure 3:**
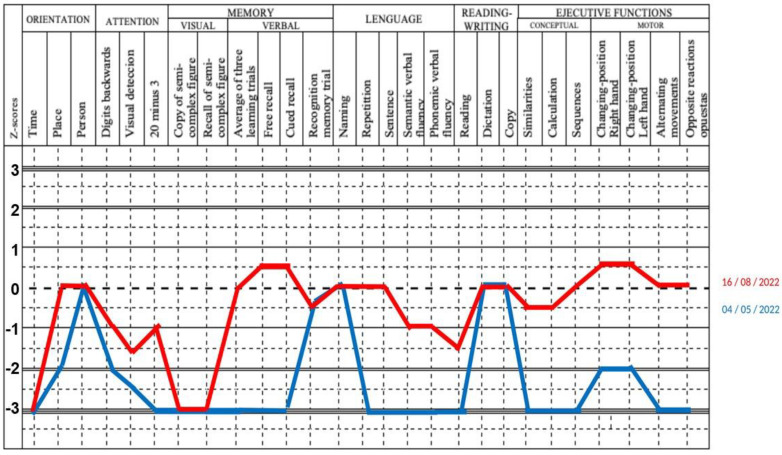
Basal and post-treatment neuropsychology assessment of patient #2. NEUROPSI modified the profile from Ostrosky’s original of the second patient, which integrated severe alterations in multiple domains with frontal-subcortical involvement (blue line) characterized by alterations in visual attention, concentration, hindering working memory, loss of working memory, and difficulty in spontaneous memories. Impairment in calculation and reading capacity, with response imitation and echolalia, with word omission and stiffness of logical thinking. Slowing of thought alteration in propositional verbal fluency, visuospatial processes with micrography, visual memory, and dysexecutive failures. After a 3-month follow-up, the reassessment showed a homogeneous cognitive profile with significant improvement (from severe to mild) in attention and concentration and constructive praxis (visuospatial-motor and processing speed) (red line).

At admission, the three DM with encephalopathy presented emotional lability, impaired attention, verbal memory, and language changes. The MoCA test demonstrated mild cognitive impairment, significantly different in comparison to healthy controls (22 points (18–25) vs. 29 points (29–30) (*p* = 0.0167) (Table 1). The neuropsychological assessment showed multiple-domain amnesic neurocognitive impairment and cortico-subcortical compromise characterized by deficits in visual attention, concentration, impaired working memory, calculation, and reading ability. Also, abnormal spontaneous and visual memories, verbal fluency, and executive dysfunction were identified (Figure 2 and Figure 3).

Ferritin levels were significantly higher in DM subjects with encephalopathy compared to healthy controls (1752 ng/mL (1357–1994) vs. 21.8 ng/mL (7.7–55.5), *p* = 0.039). Also, active DM had an increase in alanine aminotransferase (ALT) and aspartate aminotransferase (AST) in comparison with remission DM (ALT: 163 U/L (44–296) vs. 18 U/L (10–19) p = 0.039) (AST: 115 U/L (72–355) vs. 15 U/L (13–15.9) *p* = 0.039), respectively (Table 1). A myopathic pattern in electromyography was observed in all the patients with DM. Past medical history and chronic medication were irrelevant; all patients had been previously healthy. Regarding comorbidities, the anti-TIF-γ positive patient had autoimmune hyperthyroidism, and the anti-Mi2 patient presented with thrombotic microangiopathy with Coombs-negative hemolytic anemia without overlapping other autoimmune connective tissue disorders.

Under suspicion of autoimmune encephalopathy, cerebrospinal fluid (CSF) analysis showed leukocytes <5 cell/mm3, protein levels <100 mg/dL, and negative VDRL. Extended panels of autoimmune encephalitis autoantibodies performed in rat brains by IHC were negative (anti-NMDAR, AMPAR, GABABR, GABAAR, LGI1, CASPR2, mGluR1, mGluR2, mGluR5, DPPX, IgLON5, Neurexin). Only the anti-TIF-γ patient showed positive oligoclonal bands in a mirror pattern.

**Figure 4:**
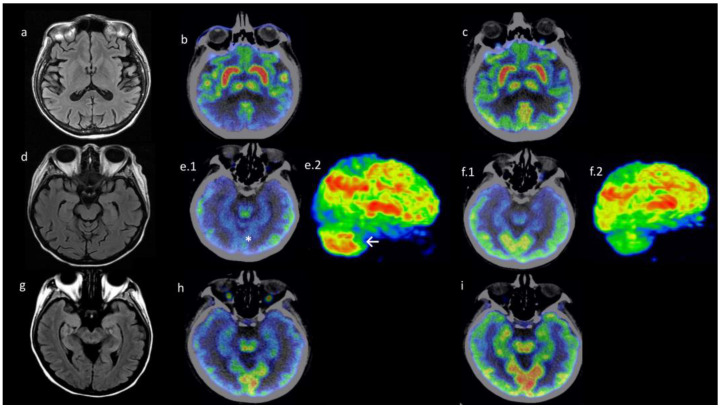
Immunological findings in MRI and PET/CT images before and after treatment. Left column: Magnetic resonance images. Central column: Pre-therapy PET/CT images. Right column: Post-therapy images. Upper row: Case #1. (**a**) FLAIR MRI does not show any abnormality. (**b**) For semiquantitative analysis with CortexID software, we found a Z-score of −7.7 and −5.4 for occipital and frontal lobes, respectively. However, the mesial temporal region and cerebellum showed Z-scores of +7.9 and +2. (**c**). After treatment, all Z-scores demonstrated better values (occipital −3.1, frontal −2.4, mesial temporal +2.8, cerebellum −0.06). Middle row: Case #2. (**d**). FLAIR MRI did not show any abnormal findings. Panels (**e.2**) and (**f.2**): FDG maximum intensity projection. Panels (**e.1**) and (**e.2**). Brain FDG PET/CT. Cortex ID showed a Z-score of −7.25 for the frontal lobe and −2.7 for the occipital. Higher positive Z-score were found in mesial temporal +2.47 and cerebellum +4.66 (*), Negative Z-score values showed improvement after therapy (frontal +2.5, occipital −1.68). Temporal mesial turned negative with a Z-score of −2.39, and cerebellum was normal (−0.4) (←), (Panels (**f.1**) and (**f.2**)). Lower row: Case #3. (**g**). FLAIR MRI with increased signal intensity in both mesial temporal lobes. (**h**) Cortex ID showed a Z-score of −7.58 for the frontal lobe and −2.17 for the occipital. Higher positive Z-score were found in the mesial temporal +5.84 and cerebellum +5.6. (**i**) FDG PET post-therapy showed normal metabolism.

**Figure 5:**
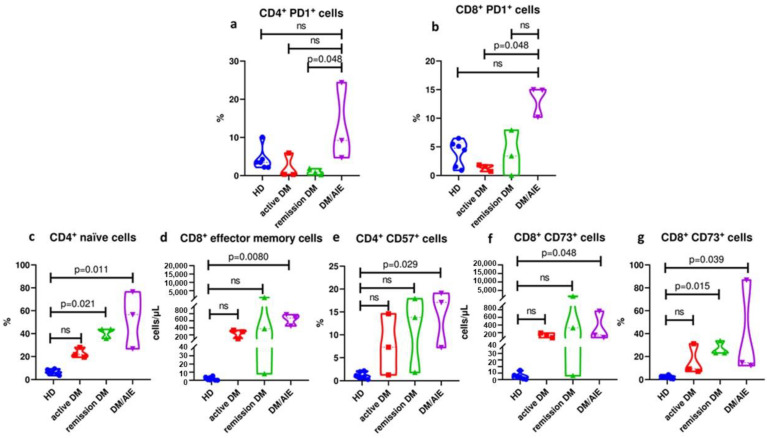
Flow cytometry immunophenotype demonstrates CD4^+^PD1^+^ and CD8^+^PD1^+^ T cell expansion in dermatomyositis/encephalopathy patients (DM/AIE) compared to remission and active DM patients. DM/AIE patients had increased naïve, effector memory and CD57^+^ and CD73^+^ T cells compared to healthy donors. (**a**,**b**) CD4^+^ PD1^+^ and CD8^+^ PD1^+^ cells are expanded in DM/AIE patients compared to remission (14.9% [10.2–15] vs. 1.58% [0.71–1.84] *p* = 0.048) and active (9.2% [4.7–24.4] vs. 0.83% [0.2–1.85] *p* = 0.048) DM patients, respectively. (**c**) Increased percentage of CD4^+^ naïve cells in DM/AIE (56.6% [26.8–76.5] vs. 6.43% [4.52–8.8] *p* = 0.011), and in remission DM (43.8% [35.8–43.8] vs. 6.43% [4.52–8.8] *p* = 0.021) compared with healthy donors. (**d**) Greater absolute numbers of CD8^+^ effector memory cells in DM/AIE were observed (340 cells/μL [228–389] vs. 0.59 cells/μL [0.18–4.2] *p* = 0.008) in compression with healthy donors. (**e**) The augmented percentage of CD4^+^ CD57^+^ cells in DM/AIE (17.1% [7.23–19.1] vs. 1.01% [0.70–2.03] *p* = 0.029) compared with healthy donors. (**f**,**g**) Absolute numbers and percentage of CD8^+^ CD73^+^ cells were higher in DM/AIE patients (139 cells/μL [72.5–764] vs. 4.26 cells/μL [1–8.56] *p* = 0.048)/(14.8% [12.3–86.9] vs. 2.26% [0.82–3.21] *p* = 0.039) in contrast with healthy donors. Also, the percentage of CD8^+^ CD73^+^ cells in the remission DM group was higher (23.2% [23.1–33.7] vs. 2.26% [0.82–3.21] *p* = 0.015) compared to healthy donors.

Due to severe axial weakness, the three patients received five doses of 1 g of IV methylprednisolone and 5–10 plasmapheresis sessions. Then, 1 g/kg of IVIg was used in anti-Mi2 patients. Maintenance therapy based on 2 g of rituximab was given. Anti-TIF-γ and anti-MDA5 positive patients showed complete clinical neurocognitive response (Figure 4 and Figure 5) and neuroimaging normalization. The third patient died secondary due to a cardiac arrest during the second administration of IVIg.

### 3.3. Neuroimaging Findings: Patients with Dermatomyositis-Related Encephalopathy Had Differential Metabolism Pattern in PET/CT Images

Remarkably, only anti-Mi2 positive patients had brain MRI and EEG abnormalities with increased bilateral T2 signal intensity of the hippocampus and left frontotemporal triphasic waves (Figure 4a,d,g).

In all three cases, central nervous system 18FDG-PET/CT showed generalized cortical hypometabolism, with hypermetabolism in the mesial temporal, cerebellar hemispheres, caudate, and putamen (Figure 4b,e.1,e.2,h).

In the visual analysis, the areas with the highest metabolism were the mesial temporal region, cerebellum, and lenticular nucleus. The areas with the lower metabolism were the occipital and frontal lobes. All these findings correlated with the CortexID software analysis. In the baseline PET evaluation, the most common areas with hypermetabolism were the mesial temporal and cerebellum (Z-score average of +5.4 and +4.09), whilst hypometabolism was found in the frontal and occipital lobes (Z-score of −6.74 and −4.19). The subsequent PET evaluation demonstrated a normalization of Z-score in the areas with hypermetabolism (mesial temporal of +2 and cerebellum +0.8). (Figure 4c,f.1,f.2,i).

### 3.4. Patients with Dermatomyositis-Related Encephalopathy Are Characterized by a Differential T Cell Profile in Peripheral Blood

CD4+PD-1+ and CD8+PD-1+ T cell expansion is the distinctive hallmark of DM subjects with encephalopathy in comparison to remission and active DM patients.

Patients with DM and encephalopathy had an increased percentage of CD4+ PD-1+ compared to remission patients with DM (14.9% [10.2–15] vs. 1.58% [0.71–1.84] *p* = 0.048) (Figure 5a). Also, these patients had a higher percentage of CD8+ PD-1+ cells in comparison to those with active DM (9.2% [4.7–24.4] vs. 0.83% [0.2–1.85] *p* = 0.048) (Figure 5b). We did not find any significant differences in PD1 expression assessed by MFI.

Naïve and effector memory T cells are increased in DM patients with encephalopathy compared to healthy controls.

In patients with DM-related encephalopathy and with DM in remission, the percentage of naïve CD4+ T cells was increased in comparison to healthy controls (HC) (56.6% [26.8–76.5] vs. 6.43% [4.52–8.8] *p* = 0.011), and (43.8% [35.8–43.8] vs. 6.43% [4.52–8.8] *p* = 0.021) respectively (Figure 5c). Also, the absolute number of CD8+ effector memory T cells was increased in DM with encephalopathy in comparison to HC (340 cells/μL [228–389] vs. 0.59 cells/μL [0.18–4.2] *p* = 0.008) (Figure 5d).

CD4+CD57+ and CD8+CD73+ T cell subsets are expanded in DM patients with encephalopathy compared to HC.

Finally, in comparison to HC, in DM patients with encephalopathy, we evidenced a higher proportion of CD57+ CD4+ T cells (CD57+ CD4+ 17.1% [7.23–19.1] vs. 1.01% [0.70–2.03] *p* = 0.029), increased absolute number and proportion of CD73+ CD8+ T cells (CD73+ CD8+ 139 cells/μL [72.5–764] vs. 4.26 cells/μL [1–8.56] *p* = 0.048), and (14.8% [12.3–86.9] vs. 2.26% [0.82–3.21] *p* = 0.039) respectively (Figure 5f,g).

Similarly, an increased proportion of CD73+ CD8+ lymphocytes in remission DM patients was documented in contrast to HC (CD73+ CD8+ 23.2% [23.1–33.7] vs. 2.26% [0.82–3.21] *p* = 0.015) (Figure 5g). No significant differences in CD57 and CD73 MFI were observed.

## 4. Discussion

Our findings indicate that patients with DM and CNS involvement, particularly encephalopathy, exhibit a distinctive pattern on cerebral 18FDG-PET/CT and a differential peripheral blood immunophenotype. These patients present with subacute cognitive changes predominantly affecting attention, working memory, and language. Immunologically, they are characterized by an expansion of CD4+PD-1+ and CD8+PD-1+ T cells compared with remission and active DM patients, respectively. Additionally, they demonstrate increased naïve and effector memory T cells, as well as enhanced CD57/CD73 expression in lymphocytes compared to healthy controls, which may contribute to the pathogenesis of neurocognitive impairment in DM. These clinical, neuroimaging, and immunological findings could help identify a subgroup of patients who may benefit from a multidisciplinary assessment and targeted immunotherapy.

CNS diseases exceptionally overlap with DM and represent an underrecognized complication. In the literature, we found a total of 6 adult case reports showing the association between DM and neuromyelitis optica, CNS vasculopathy, and encephalomyelitis [16,17]. However, encephalopathy has been rarely perceived as a potential differential diagnosis, which negatively impacts the prognosis of these patients [18].

These immune-related encephalopathies are characterized by cognitive decline with a subacute onset, rapid progression, and fluctuating course with coexisting organ-specific autoimmunity and inflammatory findings in the cerebrospinal fluid. All of these features are present in the adequate clinical, radiological, and serological autoimmune context [19]. The largest clinical series published by the Mayo Clinic Autoimmune Dementia and Encephalopathy Study Group reported 72 patients with suspected autoimmune encephalopathy, of whom 46 (64%) presented an improvement in cognitive status after immunotherapy [20].

Autoreactive B and T cell responses may be the shared pathway linking dermatomyositis and type I IFN signature with encephalopathy. The anti-MDA5 antibodies in DM are the only subtype to date associated with mood changes and encephalopathy [21]. The cellular response mediates systemic alterations in dermatomyositis patients, such as type I IFN neurotoxicity [22], small-vessel vasculitis [23], or disruption of the blood–brain barrier secondary to T cell infiltration [24], which could be the origin of CNS dysfunction seen in these patients.

The assessment of cognitive impairment through clinical examination is the first and most crucial step in the encephalopathy diagnosis. This syndrome is characterized by an onset of less than three months, with the acute or subacute appearance of deficits in working memory and other higher cortical functions. The diagnosis is made after the reasonable exclusion of the more common alternative causes, such as infectious, paraneoplastic, autoimmune, or neurodegenerative diseases [25]. We included the PET/CT images in order to identify objective alterations, commonly referred to as “immune-mediated patterns” [15].

In this regard, a possible explanation of non-inflammatory CSF findings seen in the patients is the previous multimodal treatment of severe DM clinical features (methylprednisolone, plasmapheresis, IVIg, and rituximab), which results in a lack of pleocytosis or high-grade hyperproteinorrachia.

The clinician must investigate the intake of immunosuppressants, anesthetics, and antiseizure drugs, and the initial workup should include the assessment of thyroid and liver function tests [26]. In this regard, hyperthyroidism was a confusing factor in the first patient because of the similar clinical features of the steroid-responsive encephalopathy associated with autoimmune thyroiditis (SREAT). The diagnosis is made by exclusion, and the coexistence of DM and autoimmune thyroiditis is rare, with only 11 cases reported in the literature, and none of them exhibited neurological findings [26].

In the second case, the differential diagnosis of rapidly progressive dementia was important since the patient had rapid-onset behavioral changes with Tau protein and beta-amyloid in the CSF. Tau is a neuronal microtubule-associated protein involved in the stabilization of microtubules and axonal transport [27]. The presence of tau proteins in the CSF suggests neuronal cell lysis [28]. In IFN-mediated encephalopathies, Tau has been increased during the initial phase of the disease and sometimes normalizes with the resolution of symptoms, but currently, it is unknown if the increased Tau protein levels in CSF are a reflection of diffuse neuronal damage or are part of the pathogenesis of type IFN neurotoxicity [29].

The association between thrombotic microangiopathy and DM is also rare, with only 17 reported cases [30].

Higher ferritin levels in DM with encephalopathy patients compared to HC was an interesting finding because there is relevant evidence that suggests ferritin is the most significant prognostic biomarker in dermatomyositis [31].

The main aim of this report is to augment the suspicion of the encephalopathy diagnosis in patients with DM and neurocognitive abnormalities. In this regard, it would be useful to assess neuropsychological symptoms with an objective, standardized, and reliable tool [32]. In our case series, we used the NeuroPsi, which is validated in Spanish-speaking communities and allows the brief assessment of multiple cognitive domains, such as orientation, attention, memory, language, visuoperceptual abilities, and executive functions [13]. The three patients had predominant anterograde amnesia, executive dysfunction, and language alterations, which are particularly helpful in the objective measurement of improvements after immunotherapy [20].

Currently, the immunologic abnormalities of the DM-related encephalopathy are unknown. In this regard, we described for the first time the expansion of PD-1^+^ T cells in DM-related encephalopathy patients compared to remission and active DM without encephalopathy, as well an increase in effector memory, naïve, CD57^+^ and CD73^+^ T cells contrasted to healthy controls.

In DM patients, the frequencies of circulating CD4^+^ PD-1^+^ cells are remarkably enhanced and are an accurate dermatomyositis biomarker for diagnosis and bad prognosis (AUC = 0.79) [33]. Also, this overexpression of PD-1 positively correlates with IL-6, CRP, and MMT8. PD-1^+^ T cells are involved in Th1 and Th17 polarization and exhaustion due to chronic immunological autoreactive activation, implying a recurrent highly activated subpopulation, properly known as exhaustion subset cells. Previous evidence supports the pathogenic role of PD-1^+^ cells in systemic lupus erythematosus (SLE) [34], rheumatoid arthritis [35], and active dermatomyositis, especially in patients with poor prognosis [33].

The vasculopathy involved in dermatomyositis is promoted by priming endothelial cells, which could activate mainly T effector memory cells, especially CD45RA^−^CCR7^−^CD27^−^ through CD69 stable expression, suggesting this pathway as one of the first events priming of transmigrating T cells to requirements of tissue-residency such as muscle, skin, or CNS vessels [36]. Traditionally, CD57 has been suggested as a marker of immunosenescence, but recently, it has been shown that autoreactive CD57^+^ T cells induce severe autoimmune responses. In SLE patients, CD8^+^CD57^+^ T cells and IFN- γ levels are markedly higher compared to inactive patients and correlate with the SLE disease activity index (SLEDAI) score [5,37]. However, we found significant differences in the CD4^+^ compartment, which is a non-previously described phenotype in dermatomyositis. CD73 is a 70-kDa ecto-5′-nucleotidase, which is a marker of energy subsets and suppressor responses; however, it is fundamental in the induction of EAE. The transfer of WT CD8^+^CD73^+^ T cells into *cd73*^−/−^ mice induces EAE [8]. The initial description of these immunologic abnormalities settles the ground for further experimental studies involving disturbing and resetting CNS in DM.

The limitations of this study are several, including its cross-sectional nature, our small sample size of patients with DM-related encephalopathy, and the unicentral nature of our case series, which limits the generalizability of our findings; however, the main strength of our study is that it is the first clinical, neuroimaging, and immunological effort of characterization of DM-related encephalopathy. Furthermore, we show a cluster based on clinical features and immunological parameters, such as PD-1^+^ overexpression, that can help to distinguish this presentation from active and remission DM patients without CNS involvement. Further, multidisciplinary studies are needed to assess these questions.

## 5. Conclusions

In summary, dermatomyositis-related encephalopathy is a rare neurological complication. The neurocognitive profile is characterized by anterograde amnesia, executive dysfunction, and language alterations. High ferritin levels, abnormal PET/CT CNS metabolism, and peripheral immunophenotype (PD-1+, T_EM_, CD57+, and CD73+) are some highlights that could help to recognize DM patients who are prone to developing encephalopathy symptoms.

## Figures and Tables

**Table 1:** Clinical and laboratory features.

Variable	Dermatomyositis with Encephalopathy Patients (*n* = 3) Median (IQR)	Active Dermatomyositis Patients Without Encephalopathy (*n* = 3) Median (IQR)	Remission Dermatomyositis Patients Without Encephalopathy (*n* = 3) Median (IQR)	Healthy Controls (*n* = 3) Median (IQR)	*p*-Value
Age (years)	49 (46–56)	31 (31–58)	45 (29–45)	44 (36–58)	NS
Montreal Cognitive Assessment (MoCA)	**22 (18–25)**	26 (26–27)	27 (26–29)	**29 (29–30)**	**0.0167**
Manual muscle test 8 (MMT8)	96 (80–120)	92 (90–128)	150 (150–150)	-	NS
Cutaneous dermatomyositis disease area and severity index (CDASI) acute	19 (12–20)	11 (2–29)	0 (0–0)	-	NS
Cutaneous dermatomyositis disease area and severity index (CDASI) chronic	3 (0–12)	2 (1–7)	1 (0–3)	-	NS
Creatine phosphokinase (U/L)	43 (33–17,406)	330 (26–7261)	45 (39–74)	29 (20–55)	NS
Aldolase (U/L)	11.8 (3.9–266)	17 (16–78)	3.5 (3.5–4)	-	NS
Alanine aminotransferase (U/L)	41.1 (27.8–626)	**163 (44–296)**	**18 (10–19)**	28 (21.2–34)	**0.039**
Aspartate aminotransferase (U/L)	52.1 (47.4–1130)	**115 (72–355)**	**15 (13–15.9)**	34 (30–40)	**0.039**
Lactate dehydrogenase (U/L)	347 (257–2718)	436 (363–1124)	141 (110–153)	105 (79–210)	NS
C-reactive protein (mg/dL)	15 (1.5–17)	20 (1–21)	0.15 (0.02–0.46)	1 (0.6–1.12)	NS
Ferritin (ng/mL)	**1752 (1357–1994)**	340 (100–8237)	**21.8 (7.7–55.5)**	172 (127–193)	**0.039**
Gamma-globulins (g/dL)	4 (3.1–4)	4.3 (2–4.7)	2.7 (2.5–3.1)	3.34 (1.9–3.6)	NS
CSF cells (mm^3^)	0 (0–3)	-	-	-	
CSF proteins (mg/dL)	43 (27–73.4)	-	-	-	
Myositis Specific Antibodies (MSA)	TIF1γ, MDA5 and Mi2	MDA5, Mi2 and seronegative	Mi2 (×2) and seronegative		
Methylprednisolone pulses use (%)	3 (100%)	2 (66.6%)	0		
1 mg/kg/d of prednisone use (%)	3 (100%)	3 (100%)	0		
IVIg use (%)	2 (66.6%)	2 (66.6%)	1 (33.3%)		
Plasmapheresis use (%)	2 (66.6%)	0	0		
Rituximab use (%)	2 (66.6%)	2 (66.6%)	0		
Mycophenolate mofetil use (%)	2 (66.6%)	3 (100%)	0		
Tacrolimus use (%)	0	1 (33.3%)	0		
Methotrexate use (%)	0	2 (66.6%)	2 (66.6%)		
Azathioprine use (%)	0	0	2 (66.6%)		
Hydroxychloroquine use (%)	1 (33.3%)	3 (100%)	2 (66.6%)		

Relevant clinical and laboratory profiles of encephalopathy-dermatomyositis patients.

## Data Availability

The datasets used and/or analyzed during the current study are available from the corresponding author upon reasonable request.
